# Culture-dependent and culture-independent characterization of bacterial community diversity in different types of sandy lands: the case of Minqin County, China

**DOI:** 10.1186/s12866-021-02150-0

**Published:** 2021-03-22

**Authors:** Yali Wei, Fang Wang, Jiangli Gao, Yaolong Huang, Wei Ren, Hongmei Sheng

**Affiliations:** 1 Ministry of Education Key Laboratory of Cell Activities and Stress Adaptations, School of Life Sciences, Lanzhou University, Lanzhou, China; 2 School of medicine, northwest minzu university, Lanzhou, China; 3 State Key Laboratory of Marine Resource Utilization in the South China Sea, Hainan University, Haikou, Hainan Province China

**Keywords:** Desertification, Rhizosphere, Sandy land, Bacteria community, Culture-dependent method, Culture-independent method, Minqin Desert

## Abstract

**Background:**

Minqin is suffering from a serious desertification, whereas the knowledge about its bacterial community is limited. Herein, based on *Nitraria tangutorum* and *Haloxylon ammodendron* from Minqin, the bacterial community diversities in fixed sandy land, semi-fixed sandy land and shifting sandy land were investigated by combining with culture-dependent and culture-independent methods.

**Results:**

Minqin stressed with high salinity and poor nutrition is an oligotrophic environment. Bacterial community in Minqin was shaped primarily by the presence of host plants, whereas the type of plant and sandy land had no marked effect on those, which displayed a better survival in the rhizospheres of *N. tangutorum* and *H. ammodendron*. The dominant groups at phyla level were Actinobacteria, Firmicutes, Proteobacteria, Bacteroidetes, Planctomycetes, Chloroflexi, Acidobacteria and Candidate_division_TM7. The abundance of Firmicutes with ability of desiccation-tolerance was significantly higher in harsh environment, whereas Bacteroidetes were mainly distributed in areas with high nutrient content. The abundances of Proteobacteria and Bacteroidetes were relatively high in the rhizospheres of *N. tangutorum* and *H. ammodendron*, which had more plant-growth promoting rhizobacteria. A large number of Actinobacteria were detected, of which the most abundant genus was *Streptomyces*. The physicochemical factors related to the diversity and distribution of the bacterial community were comprehensively analyzed, such as pH, electrical conductivity, soil organic matter, C/N and sand, and the results indicated that Minqin was more suitable for the growth of *N. tangutorum*, which should be one of most important sand-fixing plants in Minqin.

**Conclusions:**

The bacterial community diversities in different types of sandy lands of Minqin were comprehensively and systematically investigated by culture-dependent and culture-independent approaches, which has a great significance in maintaining/restoring biological diversity.

**Supplementary Information:**

The online version contains supplementary material available at 10.1186/s12866-021-02150-0.

## Background

Desertification is a serious global environmental problem [1–3]. China is also under the influence of the desertification, and the areas are mainly concentrated in western of northeast and northwest [4, 5]. In the northwest China, water resources are dwindling due to years of peripheral expansion by Tengger Desert and Badain Jaran Desert to the downstream Shiyang River basin; desertification in Minqin is more and more seriously [6]. Desert plants with adversity resistance can survive and reproduce in unfavorable environments, such as extreme barrenness, drought, saline-alkali and high intensity radiation, which play an important role in maintaining the ecological stability of extremely poor environments and inhibiting the continuous expansion of desertification.

Minqin desertification is one of the birthplaces of desert, encroaching the valuable living space, endangering the existence and development of humankind, and accelerating the expansion of sandstorms to the east. Many researchers have paid much attention on reforestation [7, 8], relationship between plants and soil properties [9–11], and ecological and hydrological processes [12, 13] for the prevention/restoration of desertification. It is well-known that diversity and distribution of soil microbial community vary distinctly in different ecosystems, and the variation is supposed to be related to a series of soil abiotic and biotic factors [14]. Microorganisms are key components in the proper functioning of the ecosystem by the transformation of organic carbon, sulfur, nitrogenous compounds and metals, thereby establishing vegetation and maintaining soil physical structure [15, 16]. The natural vegetation of Minqin Desert is mainly small shrubs and herbs. Among them, *Nitraria tangutorum* and *Haloxylon ammodendron* are the dominant shrubs, and their well-developed roots are conducive to the interaction between plant and microbial community, thus promoting plant growth and improving soil structure [17].

In order to better understand the role of microbes, it is important to increase our knowledge of microbial diversity in a specific environment. However, few reports were about the microbial community structures inhabiting Minqin Desert, especially what factors determine their distribution characteristics. Therefore, it is necessary to investigate the microbial community structure of Minqin Desert, thereby providing important information for desertification control. For this purpose, soil samples of rhizosphere and non-rhizosphere of *N. tangutorum* and *H. ammodendron* from fixed sandy land, semi-fixed sandy land and shifting sandy land in Minqin were collected. The culture-dependent and culture-independent methods were combined to comprehensively evaluate the diversity and distribution of bacterial communities, and preliminarily analyzed the key factors shaping the distribution of bacterial communities. This research will lay a solid foundation for maintaining/restoring biological diversity.

## Results

### Soil properties of sandy lands

The general physicochemical properties of rhizospheres of two dominant plants and bulk soils from three types of sandy lands of Minqin Desert were summarized in Table 1. The soil pH was ranged from 8.14 to 9.16. The pH was significantly different between each sampling site in the fixed sandy land, whereas the difference in other two sandy lands was not significant between their own sampling sites. There was no significant difference in electrical conductivity (EC) of non-rhizospheres from three types of sandy lands, whereas the overall trend of EC in rhizospheres was higher than that in non-rhizospheres. EC of *N. tangutorum* in fixed sandy land was significantly higher than that in other two sandy lands. Additionally, the content of soil Na^+^ was ranged from 101.18 to 444.33 mg/kg. The contents of Na^+^ from *N. tangutorum* rhizosphere in fixed and shifting sandy lands were significantly higher than that from other sites, whereas the content of Na^+^ from *H. ammodendron* rhizosphere in semi-fixed sandy land was significantly higher than that from other sites. Soil total carbon (STC) displayed significant differences between different sites (ranged from 0.293 to 0.921 g/kg), but still at a low level. Except for the rhizosphere and non-rhizosphere of *N. tangutorum* in fixed sandy land, the differences of soil total nitrogen (STN) among other sites were not significant. C/N was significantly different in *N. tangutorum* rhizosphere from different types of sandy lands, whereas there was no obvious change in other samples. The contents of soil organic matter (SOM) in the bulk soils were as follows: fixed sandy land > semi-fixed sandy land > shifting sandy land, which had a significant difference between each other. The content of SOM in rhizosphere of *N. tangutorum* from shifting sandy land was the highest (shifting sandy land > semi-fixed sandy land > fixed sandy land), whereas there was no significantly different between those in rhizospheres of *N. tangutorum* from fixed and semi-fixed sandy lands. The contents of SOM in rhizosphere of *H. ammodendron* were as follows: semi-fixed sandy land > shifting sandy land > fixed sandy land, whereas there was no significantly different between these in rhizosphere of *H. ammodendron* from semi-fixed and shifting sandy lands, and the SOM contents from *H. ammodendron* rhizosphere in semi-fixed and fixed sandy land were all more than those in control samples. Additionally, the soil texture in different sampling sites also displayed significantly different, such as sand (Table 1).

**Table 1:** Soil physicochemical properties of Minqin Desert

Sample	pH	EC	STP	STN	STC	SOM	Na^+^	K^+^	C/N	Soil texture (%)		
		(μs/cm)	(g/kg)	(g/kg)	(g/kg)	(g/kg)	(mg/kg)	(mg/kg)		Clay	Silt	Sand
AC	8.733 ± 0.050 ^c^	46.733 ± 4.000 ^ef^	0.030 ± 0.001 ^a^	0.045 ± 0.006 ^a^	0.921 ± 0.001 ^a^	0.546 ± 0.021 ^a^	190.272 ± 1.831 ^c^	210.976 ± 6.770 ^bc^	20.262 ± 2.705 ^d^	0.324 ± 0.015 ^g^	3.223 ± 0.015 ^e^	96.453 ± 0.015 ^b^
AN	8.140 ± 0.168 ^d^	240.477 ± 4.500 ^a^	0.027 ± 0.001 ^ab^	0.030 ± 0.003 ^b^	0.792 ± 0.001 ^b^	0.119 + 0.055 ^d^	338.063+ 51.706 ^b^	240.422 + 63.791 ^c^	26.005 2.462 ^bcd^	1.645 ± 0.015 ^b^	6.731 ± 0.015 ^a^	91.624 ± 0.015 ^e^
AH	9.343 ± 0.108 ^a^	50.137 ± 0.210 ^ef^	0.025 ± 0.001 ^bc^	0.025 ± 0.004 ^bc^	0.579 ± 0.001 ^f^	0.160 ± 0.025 ^d^	193.203 ± 2.459 ^c^	241.461 ± 5.129 ^b^	22.816 ± 3.395 ^bcd^	0.673 ± 0.015 ^ef^	2.771 ± 0.015 ^h^	96.556 ± 0.015 ^b^
BC	9.163 ± 0.060 ^ab^	45.247 ± 3.601 ^ef^	0.017 ± 0.001 ^g^	0.014 ± 0.002 ^f^	0.524 ± 0.132 ^o^	0.159 ± 0.013 ^d^	101.177 ± 3.522 ^d^	118.169 ± 6.338 ^d^	38.749 ± 5.673 ^a^	0.095 ± 0.015 ^h^	2.042 ± 0.015 ^i^	97.864 ± 0.015 ^a^
BN	8.843 ± 0.057 ^bc^	53.787 ± 0.700 ^e^	0.024 ± 0.001 ^df^	0.017 ± 0.002 ^def^	0.526 ± 0.001 ^io^	0.151 ± 0.026 ^d^	133.537 ± 6.769 ^cd^	120.652 ± 5.841 ^d^	31.216 ± 3.543 ^abc^	0.746 ± 0.015 ^e^	2.925 ± 0.015 ^h^	96.330 ± 0.015 ^b^
BH	9.063 ± 0.035 ^abc^	63.740 ± 1.650 ^d^	0.021 ± 0.001 ^bcdf^	0.023 ± 0.002 ^bcde^	0.568 ± 0.002 ^g^	0.333 ± 0.095 ^b^	444.330 ± 72.481 ^a^	178.639 ± 6.913 ^c^	24.824 ± 2.086 ^bcd^	0.019 ± 0.010 ^i^	2.023 ± 0.010 ^i^	97.958 ± 0.010 ^a^
CC	8.997 ± 0.086 ^abc^	51.037 ± 1.102 ^ef^	0.023 ± 0.003 ^bcd^	0.024 ± 0.001 ^bc^	0.557 ± 0.162 ^h^	0.061 ± 0.007 ^e^	174.237 ± 4.811 ^c^	97.610 ± 5.309 ^d^	23.146 ± 0.279 ^bcd^	1.280 ± 0.015 ^c^	4.575 ± 0.015 ^c^	94.144 ± 0.015 ^h^
CN	8.790 ± 0.113 ^bc^	137.447 ± 2.650 ^b^	0.020 ± 0.001 ^cdf^	0.018 ± 0.001 ^cdef^	0.293 ± 0.025 ^q^	0.295 ± 0.001 ^bc^	279.801 ± 10.332 ^b^	104.676 ± 0.287 ^d^	16.457 ± 0.202 ^d^	1.291 ± 0.015 ^c^	6.202 ± 0.015 ^b^	92.507 ± 0.015 ^i^
CH	9.063 ± 0.104 ^abc^	74.793 ± 12.400 ^c^	0.025 ± 0.001 ^cdf^	0.021 ± 0.001 ^cdef^	0.655 ± 0.003 ^c^	0.287 ± 0.039 ^bc^	190.392 ± 3.862 ^c^	186.488 ± 7.767 ^c^	31.779 ± 0.643 ^abc^	1.989 ± 0.015 ^a^	6.783 ± 0.015 ^a^	91.228 ± 0.015 ^f^

Note: The first letter in the code represents sandy types (A, fixed sandy land; B, semi-fixed sandy land; C, shifting sandy land) and the second letter in the code represents the plant species (C, bulk soil without plant; N, *N. tangutorum*; H, *H. ammodendron*). Values are means of three replicates, and means in the column followed by different letters are significantly different (*P* < 0.05). The statistical differences in data are statistically analyzed by the least-significant difference test (*P* < 0.05) using SPSS version 17.0 for Windows (SPSS Inc., Chicago, USA)

### Analysis of diversity and distribution of bacterial community by culture-dependent method

**Fig. 1:**
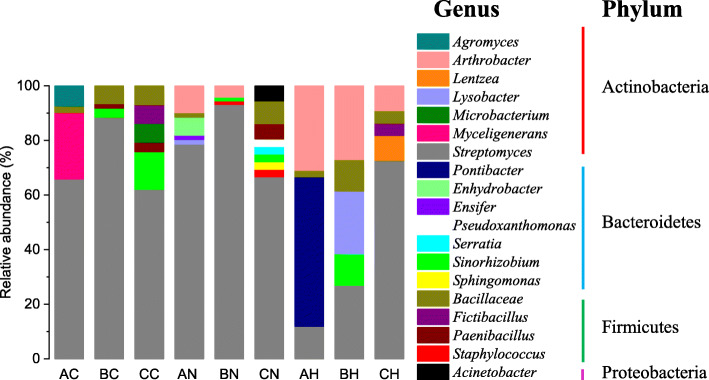
Distribution of partial sequences of culturable bacteria from bulk soils and rhizospheres of *N. tangutorum* and *H. ammodendron* at fixed sandy land, semi-fixed sandy land and shifting sandy land on the level of genus, and all genera belonged to Actinobacteria, Firmicutes, Proteobacteria and Bacteroidetes. The first letter in the code represents sandy types (A, fixed sandy land; B, semi-fixed sandy land; C, shifting sandy land) and the second letter in the code represents the plant species (C, bulk soil without plant; N, *N. tangutorum*; H, *H. ammodendron*)

Seventy-seven morphologically divergent bacterial strains were isolated with reasoner’s 2A agar (R_2_A; Table S1), and the bacterial count varied from 6.5 × 10^4^ to 4.04 × 10^7^ CFU/mL. Nineteen genera belonged to four phyla, Actinobacteria, Firmicutes, Proteobacteria and Bacteroidetes (Fig. 1). Bacterial diversity was found to be maximum (up to 80.77%) in the phylum of Actinobacteria, and the largest genus was *Streptomyces*, followed by *Pontibacter* from Bacteroidetes (5.9%) only isolated from *H. ammodendron* rhizosphere in fixed sandy land. The relative high abundance of culturable bacterial community in shifting sandy land, especially *N. tangutorum* in shifting sandy land, may give an explanation of why *N. tangutorum* shown the best growth status with the largest abundance of bacterial community. *Streptomyces*, *Arthrobacter*, *Lysobacte*, *Sinorhizobium* and *Bacillus* were isolated from the cultivable bacteria community of rhizospheres in semi-fixed sandy land, which were more balance and most of them were plant growth-promoting rhizobacteria. The relative abundance of culturable bacterial community of bulk soil in shifting sandy land was larger than that in other two types of sandy lands. However, the similarity between some bacteria and the closest species was less than 99% by BLAST analysis of the 16S rDNA gene sequences, which indicated that there may be some potential novel species (Table S1). It’s worth noting that we didn’t find observed colonies in the dark for 3 days at 28 °C by culture-dependent method, and the observed colonies can be found in the dark for 5–7 days at 28 °C. We reason that the bacteria from the desert have lower activities than that from other places.

### Analysis of diversity and distribution of bacterial community by culture-independent method

#### Overview of diversity and distribution of bacterial community

Species accumulation curve (SAC) is used to estimate the number of operational taxonomic units (OTUs) in a particular area, which is a tool used to investigate the species composition of the samples and predict their species richness. In the present study, SAC was used to determine whether the observed bacterial diversity represented the overall bacterial diversity. As shown in Fig. S1, SAC was increasing and negatively accelerated, which tended to an asymptote, indicating that the sequencing results were available, and could reflect the bacterial community structure composition of the real samples.

**Fig. 2:**
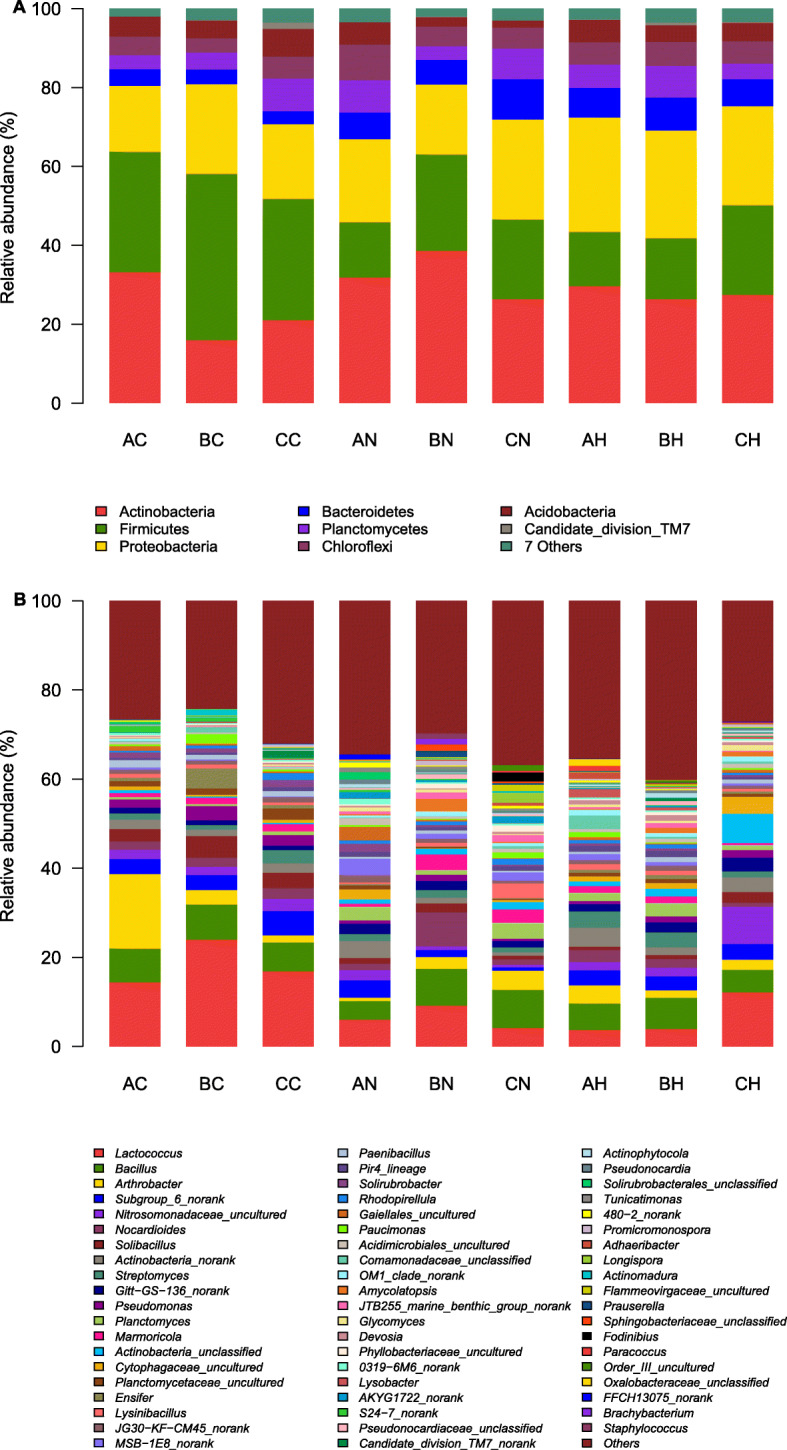
Relative abundances of the dominant bacterial groups in all soils on the level of phylum (**a**) and genus (**b**). The first letter in the code represents sandy types (A, fixed sandy land; B, semi-fixed sandy land; C, shifting sandy land) and the second letter in the code represents the plant species (C, bulk soil without plant; N, *N. tangutorum*; H, *H. ammodendron*)

In total, 382,306 quality sequences were obtained from all of the 27 samples with three replicates, and 10,505–17,126 sequences were obtained from per sample (mean = 14,159). The read lengths ranged from 396.22 to 397.15 bp with an average of 396.50 bp. The dominant groups (relative abundance > 5%) across all soil samples were Actinobacteria, Firmicutes, Proteobacteria, Bacteroidetes, Planctomycetes, Chloroflexi, Acidobacteria and Candidate_division_TM7 (Fig. 2). These groups accounted for more than 94% of the bacterial sequences, which was agreed with that from Sonoran Desert soils [18]. Reportedly, the most dominant group in Tenggeli Desert was Proteobacteria phylum [4]. Groups of Caldiserica, Candidate_division_OD1, Tenericutes, Thermotogae, WCHB1–60, Deferribacteres, Fibrobacteres, TM6, BHI80–139, Caldiserica, SHA-109, Elusimicrobia, JL-ETNP-Z39, Chlorobi and Nitrospirae displayed less abundant (relative abundance > 0.1%), but were still found in most desert soils. We found that the phylum distribution in Minqin still had a relative peculiar pattern. Actinobacteria was the most abundant phylum in most of the analyzed samples with the exception of bulk soils from semi-fixed and shifting sandy lands, whereas Firmicutes was the most abundant phylum from semi-fixed and shifting sandy lands. The relative abundance of Bacteroidetes was larger in shifting sandy land of *N. tangutorum*, unfortunately, we didn’t isolate Bacteroidetes by culture-dependent method. *Lactococcus* was accounted for the most in the bacterial community distribution of Minqin Desert, especially in non-rhizosphere soil samples, followed by *Bacillus*. According to the distribution of plants, *N. tangutorum* in shifting sandy land had higher stress resistance, whereas *H. ammodendron* had better growth status in fixed and semi-fixed sandy land.

#### Relationship between bacterial community distribution and type of sandy land and host plant

**Fig. 3:**
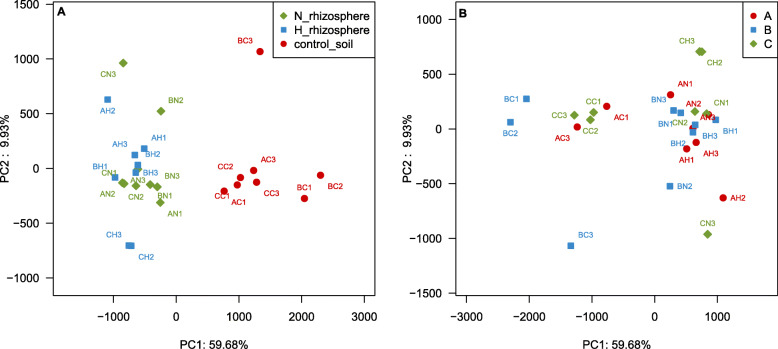
PCA of the main factors affecting bacterial community distribution. (**a**): The effects of plant species on the bacterial community distribution: control soil, bulk soils without plant; N_rhizosphere, rhizospheres of *N. tangutorum*; H_rhizosphere*,* rhizospheres of *H. ammodendron*. (**b**): The effects of types of sandy lands on the bacterial community distribution: A, fixed sandy land; B, semi-fixed sandy land; C, shifting sandy land. The first letter in the code represents sandy types and the second letter in the code represents the plant species

The results of clustering of rhizospheres and bulk soils by principal component analysis (PCA) showed that OTUs in rhizospheres and bulk soils were clearly separated, whereas there were no large differences between the OTUs of the rhizospheres of *N. tangutorum* and *H. ammodendron* (Fig. 3a). However, no significant differences were observed in the OTUs clustering of the bulk soils, although the compositions of the bulk soils from three types of sandy lands were different (Fig. 3b).

#### Similarity and difference in the distribution of bacterial community

Overall, the highly abundant bacteria in rhizospheres at the phylum level can be well clustered, whereas those in bulk soils were in separate groups. The bacterial communities of bulk soils had a large different from those of the rhizospheres of *H. ammodendron* and *N. tangutorum*.

**Fig. 4:**
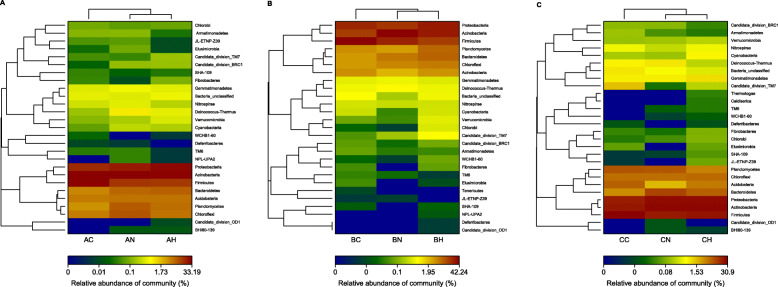
Distribution heatmap of microbial orders arranged by hierarchical clustering of samples from fixed sandy land (**a**), semi-fixed sandy land (**b**) and shifting sandy land (**c**) at the phylum level. The first letter in the code represents sandy types (A, fixed sandy land; B, semi-fixed sandy land; C, shifting sandy land) and the second letter in the code represents the plant species (C, bulk soil without plant; N, *N. tangutorum*; H, *H. ammodendron*)

In fixed sandy land (Fig. 4a), the relative abundances of BHI80–139, Chloroflexi, Deinococcus−Thermus, Proteobacteria and Bacteroidetes had large numbers in bulk soils and rhizospheres of *H. ammodendron* and *N. tangutorum*, whereas bulk soils than rhizospheres had more Firmicutes. Actually, though the relative abundances of these phyla were similar, the bacterial communities in the two rhizospheres still had a little difference. Deinococcus−Thermus as a thermophilic bacterium displayed a higher abundance in rhizosphere of *N. tangutorum*, which can be commonly isolated from arid and hot deserts.

In semi-fixed sandy land (Fig. 4b), Bacteroidetes, Acidobacteria and Chloroflexi showed higher abundance in rhizosphere of *H. ammodendron* than those in bulk soil and rhizosphere of *N. tangutorum*. Deinococcus−Thermus and Actinobacteria still displayed the largest number in rhizosphere of *N. tangutorum* in semi-fixed sandy land.

In shifting sandy land, numbers of stress-resistant bacteria were found in rhizosphere of *H. ammodendron*, such as Thermotogae and Caldiserica (Fig. 4c). It is worth noting that Deinococcus−Thermus still displayed in rhizosphere of *N. tangutorum*. Additionally, the number of Bacteroidetes was the largest in rhizosphere of *N. tangutorum*, which is often used as one of indications for good soil environment. The rhizosphere of *N. tangutorum* provided the good conductions for the survival of the Bacteroidetes. At the same time, the distribution of Acidobacteria in the rhizosphere of *N. tangutorum* was significantly higher than that in fixed and semi-fixed sandy lands. Acidobacteria could improve the pH of rhizosphere, which is beneficial to the growth of plants. In conclusion, the physiological growth state of *N. tangutorum* in shifting sandy land was much better than that in other two types of sandy lands.

#### Comparison of bacterial distribution of rhizospheres and bulk soils

**Fig. 5:**
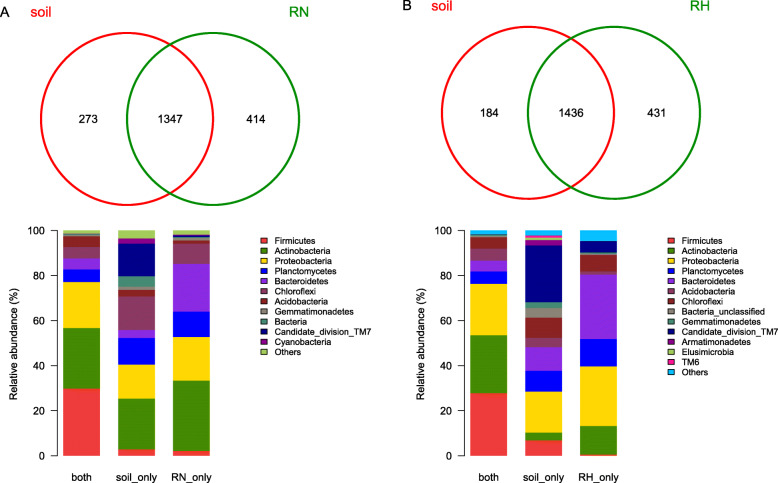
Comparison of the distribution of control samples and rhizosphere samples at the phylum level. Proportional Venn diagram showing the number of OTUs found only in the soil (soil_only), only in the rhizosphere *N. tangutorum* (RN_only) (**a**) or *H. ammodendron* (RH_only) (**b**), and in both locations. These OTUs were used to create the abundance bar graphs and the relative abundance of raw reads for different phyla belonging to the OTUs depicted under the panel Venn

As shown in Fig. 5a, of the 2034 total observed OTUs from different phyla, 1347 (66.2%) containing a proportionately large percentage of the raw reads (199,779 of the 212,390 reads or 94.1%) were found in both soil and rhizosphere of *N. tangutorum*, whereas the bulk soil and rhizosphere harbored only 273 (13.4%) and 414 (20.4%) unique OTUs, respectively. Bacteroidetes and Proteobacteria were notably abundant and disproportionately over represented in the rhizosphere of *N. tangutorum* (the bar graphs seen in Fig. 5a). Meanwhile, 2051 OTUs were found in the rhizosphere of *H. ammodendron* and bulk soils, whereas the soil and rhizosphere harbored only 184 (8.97%) and 431 (20.1%) unique OTUs, respectively (Fig. 5b). The number of OTUs of rhizosphere of *H. ammodendron* showed relative larger than that of the soil-only. However, the both soil and rhizosphere of *H. ammodendron* still had the highest number of OTUs (70.01%) with the proportionately large percentage of the raw reads (211,168 of the 218,671 reads or 96.57%). The predominate unique phylum of rhizosphere of *H. ammodendron* were Actinobacteria, Bacteroidetes and Proteobacteria, whereas the soil-only had large number of Planctomycetes. Among the bacteria in both, Firmicutes was accounted for the highest proportion.

## Discussion

### Soil physicochemical properties of Minqin Desert

The structure of bacterial communities in soil seems to be driven by the physicochemical properties of soil and the type of land [19–22]. On the order of thousands to tens of thousands of kilometers, microbial community structure is correlated to edaphic variables, such as pH, STN, SOM, moisture content and STC [23–25]. Minqin Desert is in alkaline environment. The pH of fixed sandy land was significantly affected by plants. The fixed effect of plants made fixed sandy land (less affected by wind and sand) form a relatively stable and unique micro-habitat with specific pH environment. Reportedly, pH of acidic soils almost played a decisive role in bacterial distribution [24, 26]. Additionally, the endophytic bacteria in sugarcane diversity was mainly affected by soil pH and clay [27]. The trend of EC in rhizospheres was higher than that in non-rhizospheres (Table 1), we reason that the exudates and bacteria activities of rhizosphere accelerated the decomposition of organic matter, thereby promoting the absorption of some soluble ions that were beneficial to the root system and creating a suitable osmotic pressure for the survival and reproduction of rhizosphere bacteria. Due to the severe saline-alkali stress in Minqin Desert all the year round, the desert plants and bacterial communities displayed saline-alkali tolerance and antagonistic mechanism to ensure their stable survival and genetic development. *N. tangutorum* with higher EC in fixed sandy land had a wide tolerance range for saline-alkali stress. This may be the reason why *N. tangutorum* was more adaptable to saline-alkali environment. The rhizosphere of *N. tangutorum* and *H. ammodendron* displayed high content of Na^+^ (Table 1), indicating that high content of Na^+^ didn’t limit the growth of plants lived in saline soils for long periods of time. *N. tangutorum* absorbed Na^+^ as inorganic osmotic regulator by root system, thereby adapting to saline-alkali environment [28]. Reportedly, the content of Na^+^ in Namib Desert had greatly influence on the distribution of bacteria [29]. The SOM distribution in Minqin Desert suggested that the SOM of non-rhizosphere was affected by soil crust, which showed higher SOM in fixed sand land (Table 1). SOM distribution of rhizosphere might be affected by secretion or microorganism, which was consistent with the growth state of plants. In addition, the contents of SOM were significantly different between each type of sandy land, and between those in rhizospheres of *N. tangutorum* and *H. ammodendron* (Table 1). Our research matched the resource island hypothesis in semi-fixed and shifting sandy land, which depended on the growth status of the shrubs. The decrease of shrub coverage and productivity maybe due to the decrease of deep soil water content [9, 30, 31], which gave a strong evidence for that why *N. tangutorum* under long closed fences is still at risk of death on fixed sand. Overall, Minqin Desert was stressed with high salinity and poor nutrition, which was an oligotrophic environment.

### Detection of bacterial community of rhizosphere and bulk soils at different habitats by culture-independent method

Independent techniques provide an opportunity to study a much broader spectrum of microorganisms in the rhizosphere, which can be used for detecting structure and function of rhizosphere communities. The dominant groups in Minqin included Actinobacteria, Firmicutes, Proteobacteria, Bacteroidetes, Planctomycetes, Chloroflexi, Acidobacteria and Candidate_division_TM7 according to the results of culture-independent sequencing (Fig. 2), which were similar to that in Sonoran Desert, Tenggeli Desert and Brazilian Caatinga Biome [4, 18, 32]. A high abundance of Proteobacteria has previously been associated with high availability of carbon [33–35]. Acidobacteria can well grow in oligotrophic conditions [36] and is an indicator of the acidic conditions [37, 38]. Minqin Desert is stressed with poor nutrition, and its soil pH displayed significant differences in different sites (Table 1), which maybe the justification that the actinomycetes population varied as per sampling site. Therefore, Actinobacteria and Firmicutes with high abundance in Minqin as the certain bacterial phyla, similar to Proteobacteria and Acidobacteria, may be used as indicators of nutrient status owing to their lifestyles. Additionally, some phyla related to heat resistant and documented radiotolerance, such as Thermotogae, Caldiserica and Deinococcus-Thermus, were detected in Minqin Desert. Generally, the bigger proportion of Bacteroidetes, the more fertile of soil. The phylum of Bacteroidetes is always dominant part in some black glebes, even in semi-moist desert [26], and the abundance of this phylum in Minqin Desert also displayed relatively high. The abundances of Proteobacteria and Bacteroidetes were relatively high in the rhizosphere of *N. tangutorum* from shifting sandy land and *H. ammodendron* from semi-fixed sandy land, and the both shrubs showed the best growth status in Minqin. We reason that Bacteroidetes and Proteobacteria have more plant-growth promoting rhizobacteria, and these species were the selection effect of root exudates in rhizosphere. All of these results indicated that shrubs could change bacterial community composition, and the bacterial community in rhizosphere showed a better survival.

The proportion of *Lactococcus* and *Bacillus* maybe play an important role in soil restoration, which can promote the formation of polyglutamic acid as a natural protective biofilm for soil, thereby preventing the loss of water and fertilizer. The organic acids and enzymes were synthesized by these bacterial strains, which could furtherly promote the growth of plants by the decomposition of soil macromolecules. In addition to *Lactococcus* and *Bacillus*, *Arthrobacter* known as growth-promoting bacterium also occupied the majority proportion. In our research on plant endophytic bacteria in Minqin Desert, *Lactococcus* displayed the highest abundance in the bacterial community distribution, and *Bacillu*s and *Arthrobacter* were also widely distributed. *Streptomyces* in bulk soil from shifting sandy land with relatively high content was consistent with our results of culturable bacteria. Interesting, *Fodinibius* was only distributed in *N. tangutorum* rhizosphere from shifting sandy land in Minqin Desert, which is mostly distributed in ocean and salt lakes. Therefore, we reason that the presence of *Fodinibius* played a certain role in the saline-alkali stress tolerance of *N. tangutorum*.

The total number of bacteria in rhizosphere was higher than that in non-rhizosphere, and the number of unique bacteria in rhizosphere was still higher than that in non-rhizosphere according to the results obtained by culture-independent method, indicating that the presence of host plants could promote the increase of the number of bacterial communities, whereas sandy land type was the secondary factor affecting the microbial communities in Minqin sandy lands (Figs. 3 and 4). This result was significantly different with that of the bacterial community structure of the two pine species in a semiarid ecosystem [39]. Some researchers believed that the plant type was the dominate factor for the bacterial community distribution [40, 41], whereas some others showed that the soil type was more important for that [42]. The bacteria in both accounted for a large proportion (Fig. 5), indicating that the bacterial community in the same area had a high level of cross-species homology. The distribution of bacterial community is usually affected by complex biological factors and series of abiotic factors. Due to the influence of special climatic characteristics and geographical environment, the growing plants in different study areas are also usually significantly different.

### Detection of bacterial community of rhizosphere and bulk soils at different habitats by culture-dependent method

Cultivation-based methods only address the culturable bacteria [43], which are considered to represent only a small proportion (0.1–10%) of the total bacteria presented in soil and rhizosphere [44]. Although the cultivable way can’t obtain all of the bacterial groups, it also fully predicts the dominant group in the bacterial community [43]. According to the results of high through-put sequencing, Actinobacteria, Firmicutes and Proteobacteria were most abundant phyla, which was similar to that obtained by cultivable way. The R_2_A medium was more suitable for the growth of Actinobacteria. We reason that spores produced by Actinobacteria were easier to spread, and the production of aerial hyphae and antibiotics can promote the absorption of nutrient in the medium and inhibit the growth of other bacteria. Therefore, the most obtained bacterium in the isolates was Actinobacteria, and the most abundant genus was *Streptomyces* known as a type of bacteria that can produce antibiotics. Unfortunately, *Streptomyces* was only isolated by cultivable way. It is worth to notice that the most dominant culturable bacteria of *H. ammodendron* rhizosphere was *Pontibacter korlensis* (Fig. 1), which was a kind of radiation and drought resistant bacteria belonged to Ponibacter, and *Pontibacter korlensis* has been isolated from Xinjiang, China [45]. In the culturable process, due to the limitation of preference of bacteria and nutrients in the culture, some bacteria may grow rapidly and become the dominant groups. Unfortunately, the vast majority of soil microbes are recalcitrant to culturing techniques [46], thereby preventing accurate examination of the diversity and degree of influence between biotic and abiotic factors on soil microbial communities.

Currently, the research methods of plant-associated microbiome mainly include the traditional culture-dependent method [47, 48] and culture-independent method (especially high-throughput sequencing) [49, 50]. Although only less than 1% bacteria of the microbial community could be detected by culture-dependent approach, the living bacteria can be obtained, which is an essential method for various researches [43, 51]. Surveys of bacterial communities in soil by high-throughput sequencing have illuminated the important edaphic and biotic factors on microbial diversity [18]. Culture-dependent and culture-independent approaches, despite their own limitations in measuring the diversity of microbial community, still give a reasonably great idea of microbial community structure [52]. In this work, the culture-dependent and culture-independent approaches were combined to systemically study on the bacterial community distribution in Minqin (Fig. S2), which gave a reasonably good idea of microbial community structure. A large number of potential plant growth-promoting bacteria were isolated from Minqin Desert. Further examination of the Minqin Desert microbiome is likely to reveal additional candidates for promoting plant growth in challenged environments. Culturable methods should be continuously improved and mainly used in screening functional strains, such as *Nocardiopsis quinghaiensis* isolated from high salt conditions in Qinghai, China [53] and *Pontibacter salisaro* isolated from a clay tablet solar saltern in Korea [54], which provide our future study on screening species-specific bacteria for plant growth-promoting or salt-tolerant. High-throughput way could be used to understand the distribution of bacterial communities, whereas cultivable way could be mainly used for the isolation of special strains.

### Correlation analysis between the bacterial communities and environmental factors

**Fig. 6:**
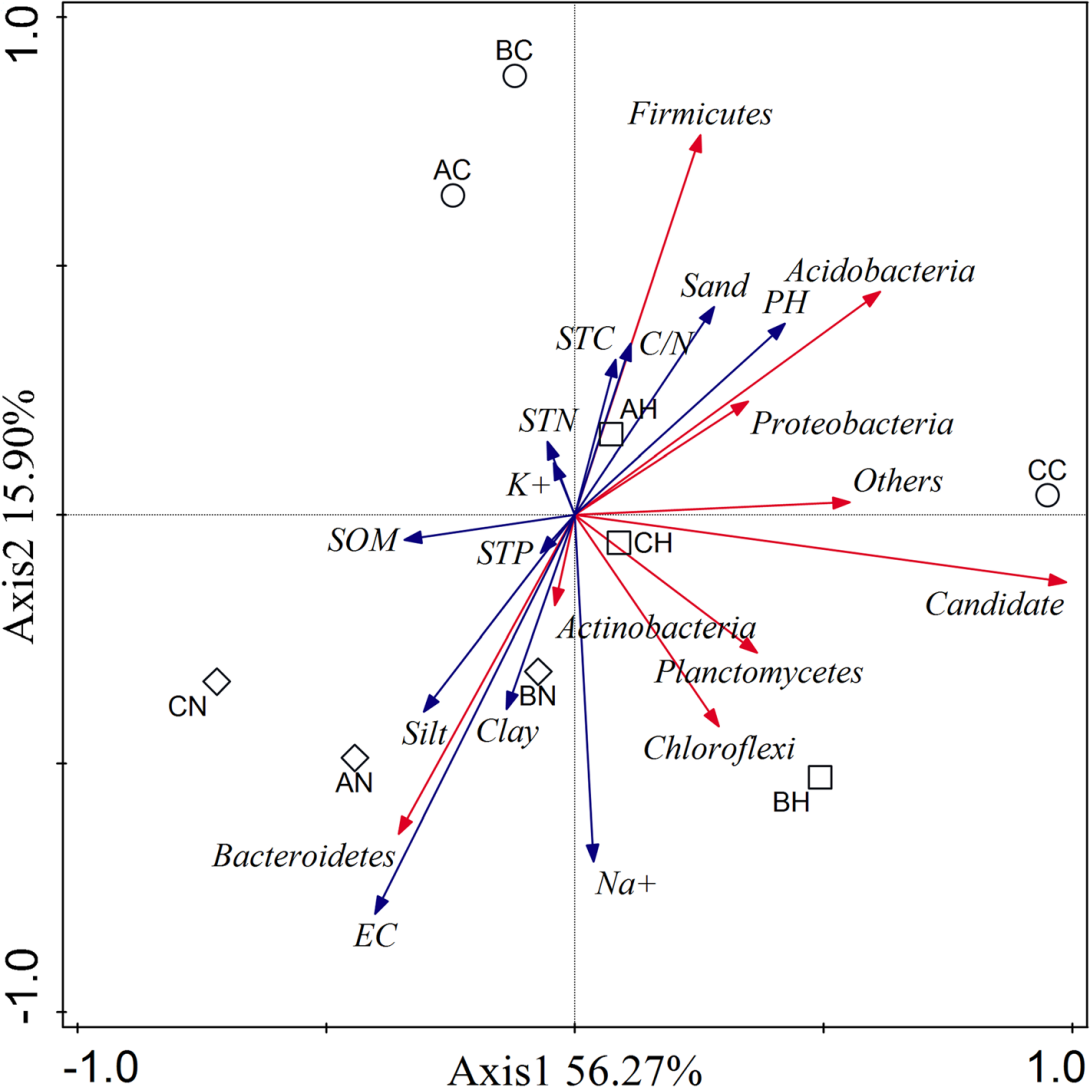
Redundancy analysis between the bacterial communities and the environmental factors present in the rhizosphere soil and bulk soil in three types of sandy lands. The first letter in the code represents sandy types (A, fixed sandy land; B, semi-fixed sandy land; C, shifting sandy land) and the second letter in the code represents the plant species (C, bulk soil without plant; N, *N. tangutorum*; H, *H. ammodendron*)

According to the physicochemical characteristics of the soil in Minqin, the distribution of the main bacteria species was analyzed. As shown in Fig. 6, the rhizosphere of *N. tangutorum* had high SOM, and the available STC of plants and bacteria was at a relatively high level. Additionally, clay and silt in soil composition also maintained at a high level. These results indicated that the microhabitat of *N. tangutorum* rhizosphere had been greatly improved, which was a relatively harsh extreme desert environment. At the same time, the higher content of Bacteroidetes also revealed the same trend, which is a sign that the corresponding soil is suitable for biological survival. Minqin was a nutrient-poor region, STN, STC, soil total phosphorus (STP) and K^+^ were all at a low level from each site. Therefore, these nutrients were not the main reason for differences in bacterial communities in Minqin.

The condition with higher sand and pH were more suitable for the growth of Firmicutes, and Firmicutes distributed in non-rhizosphere soils can adapt to the harsh environment. Therefore, in order to adapt to saline-alkali environment, Firmicutes is the main pioneer bacteria. With the selection of plant rhizosphere and the change of rhizosphere microenvironment, the proportion of Firmicutes will gradually decrease, and the bacterial diversity will also gradually increase, thereby forming a stable bacteria community structure of perennial rhizosphere. Due to the long-term selection and adaptation, the proportion of some plant associated bacteria, especially the bacteria promoting plant growth, gradually increased, and the rhizosphere bacteria gradually formed their own unique spatial distribution. Therefore, the interaction between bacteria and plants can effectively improve the soil microenvironment and increase the available nutrients in the soil, which is better for the plant growth. Such a virtuous cycle puts forward a feasible scheme for desertification control in the future, which can be applied to the effective control of desertification by artificially adding some promoting agents to promote plant growth and accelerate the improvement and restoration of microenvironment. *N. tangutorum* compared with *H. ammodendron* can more obviously improve rhizosphere environment. Therefore, we reason that Minqin sandy land is more suitable for the growth of *N. tangutorum*, which should be one of most important sand-fixing plants in Minqin.

The physicochemical factors that affect the distribution of bacteria in different environments are different [43]. The physicochemical factors related to the samples in this study were pH, EC, SOM, C/N and sand. Reportedly, the main factors affecting the distribution of bacteria in the desert were pH, total carbon, C/N and sand, whereas total nitrogen was not a key factor [18]. Nacke and coworkers showed that the main factors affecting the distribution of bacteria were organic carbon content and total nitrogen content in grassland [55]. In Minqin Desert, the rhizosphere of *N. tangutorum* had higher Na^+^ concentration and EC, which may be closely related to the halophilic mechanism of *N. tangutorum*; the composition of soil particles greatly differed between non-rhizosphere and rhizosphere, indicating that with the growth of plants, the proportion of sand particles decreased, and the phenomenon in rhizosphere of *N. tangutorum* was more obvious; the pH of non-rhizosphere soil was higher than that of rhizosphere, indicating that soil alkalinity was reduced under the effect of vegetation. SOM, TOC and C/N were significantly related to the bacterial community structure in the bacterial community in Changbai Mountain [56]. Reportedly, the bacterial community structure was mainly affected by carbon content in extreme environments, whereas total nitrogen was not the main factor [56].

Firmicutes, the main phylum adapted to barren and harsh environments, had a very significant positive correlation with sand and pH (Fig. 6), indicating that Firmicutes was the most dominant bacterial phylum in an alkaline environment. Bacteroides phylum is often regarded as one of soil quality indicator bacteria and indicates restoration in local desert. Bacteroides was densely distributed in rhizosphere of *N. tangutorum*, which was significantly positively correlated with EC, clay and silt, indicating that *N. tangutorum* had an important significance for desert restoration, and the interaction between plants and bacteria also could promote the improvement and restoration of desert in Minqin.

## Conclusions

In the present study, based on two dominate plants, *N. tangutorum* and *H. ammodendron*, twenty-seven samples with three replicates from fixed sandy land, semi-fixed sandy land and shifting sandy land in Minqin Desert were collected to investigate the distribution and diversity of bacterial community by culture-dependent and culture-independent methods. Bacterial community of Minqin Desert was shaped by the presence of host shrubs, whereas the type of the host and sandy land had no significant effect on that. The key factors, pH, EC, SOM, C/N and sand, related to the distribution of the bacterial communities were preliminary analyzed, of which EC, clay and silt were significantly positively correlated with the distribution of Bacteroides. We also found a large number of strains with specific functions and demonstrated that the important role of *N. tangutorum* in desertification restoration. For example, *Lactococcus*, *Bacillus* and *Arthrobacter* known as growth-promoting bacterium occupied majority proportion; the abundances of Proteobacteria and Bacteroidetes were relatively high in the rhizospheres of two shrubs, which had more plant-growth promoting rhizobacteria; the presence of *Fodinibius* played a certain role in the saline-alkali stress tolerance of *N. tangutorum*. This study may help the development of a strategy for revegetation management in desertified lands.

## Methods

### Study site description and sample collection

**Fig. 7:**
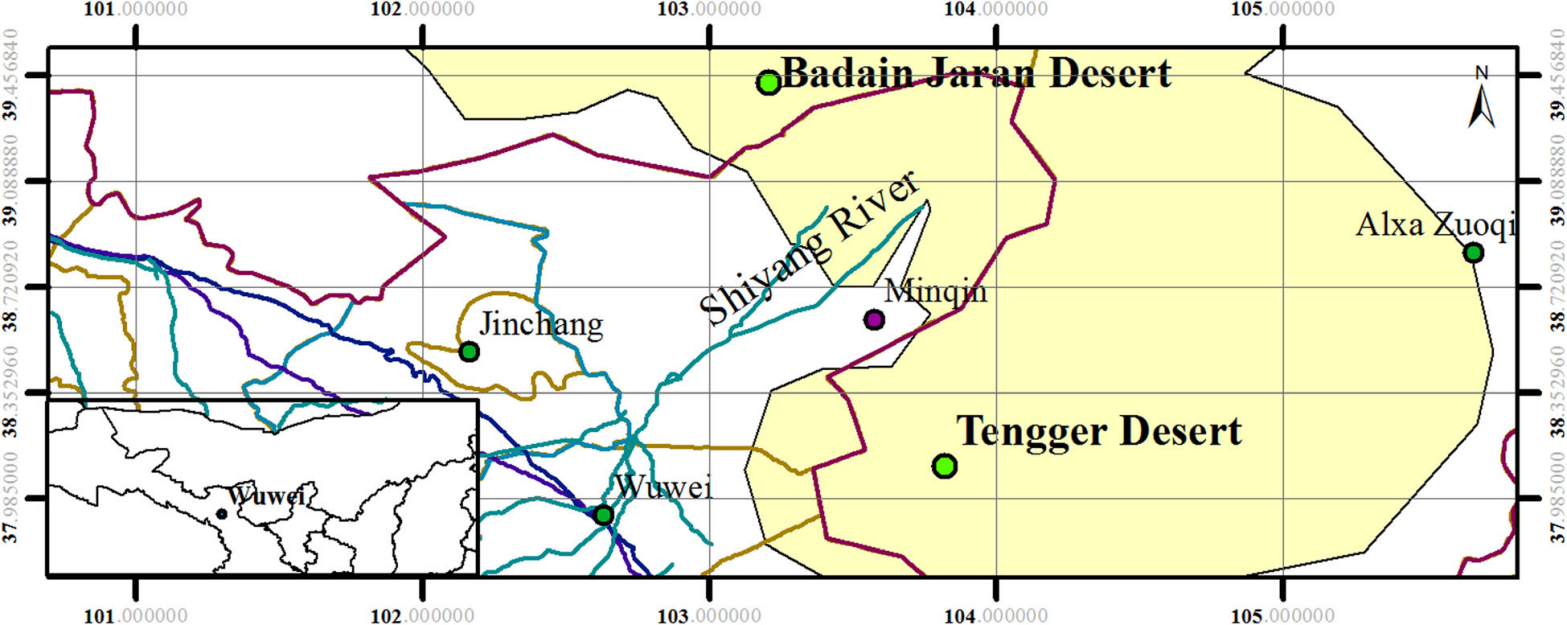
Location of the study area, Minqin County, China

Minqin, which is surrounded by the Badain Jaran Desert and the Tengger Desert, located at the lower reaches of Shiyang River next to Wuwei, Gansu Province, northwest China. The special geographical position is shown in Fig. 7. All soil samples were collected from Minqin County (38^o^62’N, 103^o^08’E). Two dominant desert plants distributed in fixed sandy land, semi-fixed sandy land and shifting sandy land, *N. tangutorum* and *H. ammodendron*, were selected for further study in Minqin. Bulk and rhizosphere soil samples were collected from *N. tangutorum* and *H. ammodendron* at maturity growth stage in three types of sandy lands. The rhizosphere soil was extracted at a soil depth of 50 cm deep along the roots, and loosely adhering soil was collected as rhizosphere soil [57]. Total 27 samples from 9 sites with three replicates were collected. Each replicate rhizosphere soil sample (approximately 200 g from each individual) was from three randomly selected adhering soil samples separated from three plants of same type at a distance of 30 to 50 m between single plants. Bulk soil as the control group, collected from each sandy land without any plant. Each replicate bulk soil sample was from three randomly selected soil samples collected from at a distance of about 200 cm from the sampling plants. All of the samples were without roots and from the same depth. The aluminium boxes were used for storing the samples at − 20 °C. All samples were divided into two parts, one for soil physical and chemical properties determination, and the other for bacteria isolation and soil DNA extraction.

### Soil physicochemical properties determination

Twenty-seven soil samples with three replicates from 9 sites were collected for analyzing soil physicochemical properties. Soil pH and EC were measured at a soil to water ratio of 1:5 (w/v) [25]. STC and STN were determined by Elemental Analyzer (Vario EL III produced by Elementar Analysen System GmbH, Langenselbold, Germany). SOM was determined using the K_2_Cr_2_O_7_-H_2_SO_4_ oxidation method of Walkley-Black [58]. STP was measured by the phosphoric acidmolybdenum-antimony colorimetric method [59]. K^+^ and Na^+^ were determined by flame photometry. Soil texture were measured by Malvern Mastersizer 2000 Laser Particle Size Analyzer [60].

### Isolation of cultural bacteria

For the isolation of soil bacteria, 5 g soil from each sample was suspended in 45 mL sterile phosphate-buffered saline (PBS), and agitated on a rotary shaker (250 rpm) for 30 min at 28 °C. The cultural conditions were chosen based on the ability to support the growth of the most abundance bacteria communities. Four gradient dilutions (10^− 3^, 10^− 4^, 10^− 5^ and 10^− 6^) were incubated on R_2_A [61] in the dark for 5–7 days at 28 °C, which was finally ascertained by preliminary selection for the condition of cultural bacteria. Colony-forming unit (CFU) per gram of soil dry mass was used to estimate the number of bacteria colonies. The distinct single strain was characterized by streaking methods to get pure colony on the basis of colony morphology analysis, which was further purified three times on R_2_A agar. All isolates were stored at − 80 °C in the R_2_A liquid media containing 20% glycerol for further use.

### DNA extraction and polymerase chain reaction (PCR) amplification

Culturable bacteria colonies were used for DNA extraction by CTAB procedure [62, 63]. The 16S rDNA genes were amplified from genomic DNA by polymerase chain reaction (PCR, GeneAmp 9700, ABI, USA) using the universal primers 27F (5′-AGAGTTTGATC(A/C)TGGCTCAG-3′) and 1492R (5′-GG(C/T)TACCTTGTTACGACTT-3′) [64]. PCR amplifications were performed using the following conditions: initial denaturation of template DNA (95 °C for 3 min), then 1 cycle consisting of denaturation (30 s at 94 °C), annealing (30 s at 55 °C), extension (1 min 40 s at 72 °C), 34 cycles at 94 °C for 30 s, 55 °C for 30 s, 72 °C for 1 min 40 s, and a final extension at 72 °C for 5 min. The culturable bacteria were identified by the 16S rDNA genes, and bacteria sequences obtained by culture-depended approach were compared with 16S rDNA reference gene sequences by BLAST (http://eztaxon-e:ezbiocloud.net). An equal volume of sterile deionized water in each batch of the PCRs as a negative control. PCR products were checked electrophoretically in a 0.4% agarose gel. The sequencing work was performed at Beijing Genomics Institute, Beijing, China.

### Illumina MiSeq sequencing for culture-independent identification

Total DNA of bacteria community was extracted from soil samples (0.5 g wet weight) with E.Z.N.A Soil DNA kit (Omega Bio-tek, Norcross, GA, US). The extracted DNA was diluted in TE buffer (10 mM Tris-HCl, 1 mM EDTA, pH 8.0) and stored at − 20 °C for further use. An aliquot (50 ng) of purified DNA from each sample was used as template for amplification. We used the 515F (5′-GTGCCAGCMGCCGCGG-3′) and 907R (5′-CCGTCAATTCMTTTRAGTTT-3′) primers to amplify the bacterial 16S rRNA V4–V5 fragments. The barcode-tagged 16S rRNA V4–V5 PCR products were pooled with other samples and sequenced by Illumina Miseq PE250 (Majorbio BioPharm Technology Co., Ltd., Shanghai, China). Every sample was amplified in triplicate with 50 mL reaction under following conditions: 30 cycles of denaturation at 94 °C for 30 s, annealing at 55 °C for 30 s, extension at 72 °C for 30 s, and a final extension at 72 °C for 10 min. PCR products checked electrophoretically in a 2% agarose gel were purified by AxyPrep DNA gel extraction kit (Axygen Biosciences, Union City, CA, USA) and quantified using QuantiFluor™-ST (Promega, USA) according to the manufacturer’s protocol. Purified amplicons were pooled in equimolar and paired-end sequenced (2 × 300 bp) using an Illumina MiSeq platform (Illumina, San Diego, CA, USA) according to the standard protocols of the Majorbio Bio-Pharm Technology Co. Ltd. (Shanghai, China). The pyrosequencing data were analyzed by QIIME version 1.5.0 software pipeline [65]. The raw reads were deposited into the NCBI Sequence Read Archive (SRA) database (Accession Number: SAMN17227076 - SAMN17227102).

### MiSeq sequencing data processing and analysis

All unique sequences from culture-dependent and culture-independent methods were used to define the number of OTUs with a 97% similarity cut-off using UPARSE (version 7.1 http://drive5.com/uparse/). A randomly selected subset of 10,000 sequences per sample for subsequent community analysis was used to get more reliable survey data. The weighted UniFrac distances calculated for the total community analyses were visualized using non-metric multidimensional scaling (NMDS) plots as implemented in PRIMER V6 [66]. Canonical correspondence analysis were used to identify the abiotic factors, most important to bacterial community composition, and they were used to construct the sediment property matrix for variation partitioning analysis with Canoco 5.03.LEfSe [67, 68]. NMDS dissimilarity matrix of R Package Version 2.0–10 was applied using the “picante” and “vegan” packages in the *R* environment [69]. The data is statistically analyzed by factor analysis, reliability analysis, one-way ANOVA, and Pearson’s product-moment correlation using SPSS 17.0 for Windows (SPSS Inc., Chicago, USA). A *P* value less than 0.05 is statistically significant. PCA was performed by CANOCO 4.5 software (Wageningen University and Research Centre, Wageningen, the Netherlands).

## Supplementary Information

**Additional file 1: Figure S1.** Species accumulation curve. **Figure S2.** Comparison of the relative abundance of the samples through high through-put technology and cultivable way. **Table S1.** Genetic diversity of culturable plant-associated bacteria from different sites.

## Data Availability

All data generated or analyzed during this study are included in this published article [and its supplementary information files]. The raw reads of pyrosequencing data have been deposited into the NCBI Sequence Read Archive (SRA) database repository, [https://www.ncbi.nlm.nih.gov/sra] and [Accession Number: SAMN17227076 - SAMN17227102].

## Abbreviations

*N.tangutorum*: *Nitraria tangutorum*; *H. ammodendron*: *Haloxylon ammodendron*
EC: Electrical conductivity
STC: Soil total carbon
STN: Soil total nitrogen
SOM: Soil organic matter
STP: Soil total phosphorus
AC: Bulk soil without plant from fixed sandy land
BC: Bulk soil without plant from semi-fixed sandy land
CC: Bulk soil without plant from shifting sandy land
AN: *N. tangutorum* rhizosphere in fixed sandy land
BN: *N. tangutorum* rhizosphere in semi-fixed sandy land
CN: *N. tangutorum* rhizosphere in shifting sandy land
AH: *H. ammodendron* rhizosphere in fixed sandy land
BH: *H. ammodendron* rhizosphere in semi-fixed sandy land
CH: *H. ammodendron* rhizosphere in shifting sandy land
CFU: Colony-forming unit
SAC: Species accumulation curve
OTUs: Operational taxonomic units
PCA: Principal component analysis; PBS: Phosphate-buffered saline
NMDS: Non-metric multidimensional scaling
