# Supplementary material for: Culture-dependent and culture-independent characterization of bacterial community diversity in different types of sandy lands: the case of Minqin County, China
Source: BMC Microbiol. 2021 Mar 22;21:87. doi: 10.1186/s12866-021-02150-0 (PMC7986352; doi:10.1186/s12866-021-02150-0)
Supplement: Supplementary file 1 — Additional file 1: Figure S1. Species accumulation curve. Figure S2. Comparison of the relative abundance of the samples through high through-put technology and cultivable way. Table S1. Genetic diversity of culturable plant-associated bacteria from different sites. [file 12866_2021_2150_MOESM1_ESM.doc]

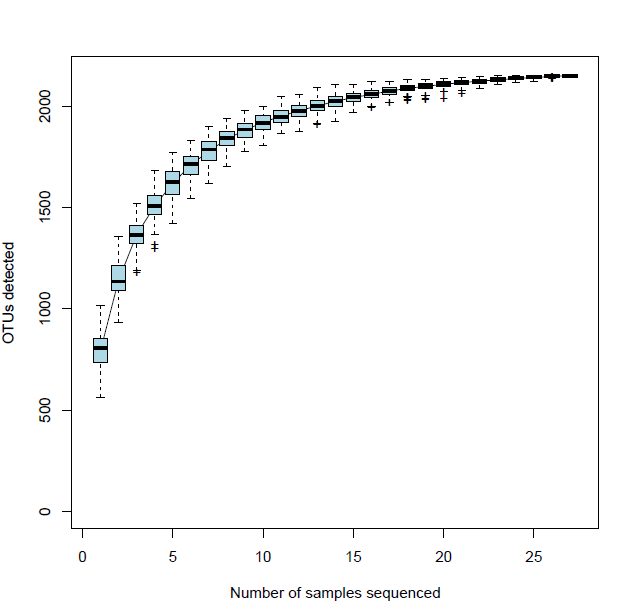


**Figure S1.** Species accumulation curve.

**Figure S2.** Comparison of the relative abundance of the samples through high through-put technology (left bar in each group) and cultivable way (right bar in each group).

Table S1. Genetic diversity of culturable plant-associated bacteria from different sites.

| Sample ID | Nearest Phylogenetic neighbor | Nearest Phylogenetic neighbor Accession No. | Similarity (%) | Phylogenetic group |
| --- | --- | --- | --- | --- |
| AC-1 | *Streptomyces coeruleofuscus strain NBRC 12757* | AB184840 | 99% | *Actinobacteria* |
| AC-2 | *Streptomyces chromofuscus strain NBRC 12851* | AB184194 | 98% | *Actinobacteria* |
| AC-3 | *Streptomyces coeruleofuscus strain NBRC 12757* | AB184840 | 99% | *Actinobacteria* |
| AC-4 | *Myceligenerans salitolerans strain XHU 5031* | JX316007 | 99% | *Actinobacteria* |
| AC-5 | *Agromyces fucosus strain NBRC 15781* | KC139245 | 98% | *Actinobacteria* |
| AC-6 | *Bacillus niacini strain NBRC 15566* | AB680904 | 98% | *Firmicutes* |
| AC-7 | *Streptomyces albogriseolus strain NBRC 3413* | AB184767 | 99% | *Actinobacteria* |
| AN-1 | *Enhydrobacter aerosaccus strain G* | AJ550856 | 99% | *γ-Proteobacteria* |
| AN-2 | *Lysobacter niabensis strain GH34-4* | DQ462461 | 97% | *γ-Proteobacteria* |
| AN-3 | *Streptomyces costaricanus strain NBRC 100773* | AB249939 | 99% | *Actinobacteria* |
| AN-5 | *Streptomyces peucetius strain NBRC 100596* | AB249907 | 99% | *Actinobacteria* |
| AN-6 | *Bacillus simplex strain LMG 11160* | AJ628743 | 99% | *Firmicutes* |
| AN-7 | *Arthrobacter tumbae strain LMG 19501* | AJ315069 | 99% | *Actinobacteria* |
| AN-8 | *Streptomyces laurentii strain NBRC 15422* | AB184669 | 98% | *Actinobacteria* |
| AN-10 | *Ensifer adhaerens* | CP007236 | 99% | *α-Proteobacteria* |
| AH-1 | *Arthrobacter siccitolerans strain 4J27* | GU815139 | 99% | *Actinobacteria* |
| AH-4 | *Pontibacter salisaro strain HMC5104* | FJ903180 | 97% | *Bacteroidetes* |
| AH-6 | *Arthrobacter tumbae strain LMG 19501* | AJ315069 | 99% | *Actinobacteria* |
| AH-7 | *Streptomyces africanus strain NBRC 101005* | AB249955 | 99% | *Actinobacteria* |
| AH-8 | *Bacillus subtilis subsp. spizizenii strain ATCC 6633* | AB018486 | 99% | *Firmicutes* |
| BC-1 | *Streptomyces coeruleofuscus strain NBRC 12757* | AB184840 | 99% | *Actinobacteria* |
| BC-3 | *Streptomyces coeruleofuscus strain NBRC 12757* | AB184840 | 99% | *Actinobacteria* |
| BC-4 | *Streptomyces curacoi strain NBRC 12761* | AB184841 | 99% | *Actinobacteria* |
| BC-6 | *Sinorhizobium meliloti 1021 strain 1021* | AL591688 | 99% | *α-Proteobacteria* |
| BC-7 | *Bacillus subtilis subsp. spizizenii strain ATCC 6633* | AB018486 | 99% | *Firmicutes* |
| BC-9 | *Bacillus litoralis strain SW-211* | AY608605 | 98% | *Firmicutes* |
| BC-10 | *PaeniBacillus xylanilyticus strain XIL14* | AY427832 | 99% | *Firmicutes* |
| BN-1 | *Streptomyces coeruleofuscus strain NBRC 12757* | AB184840 | 99% | *Actinobacteria* |
| BN-2 | *Streptomyces shaanxiensis strain CCNWHQ 0031* | FJ465151 | 99% | *Actinobacteria* |
| BN-3 | *Streptomyces tuirus strain NBRC 15617* | AB184690 | 99% | *Actinobacteria* |
| BN-4 | *Streptomyces shaanxiensis strain CCNWHQ 0031* | FJ465151 | 99% | *Actinobacteria* |
| BN-5 | *Arthrobacter globiformis strain JCM 1332* | AB089841 | 99% | *Actinobacteria* |
| BN-6 | *Streptomyces caeruleatus strain GIMN4.002* | GQ329712 | 99% | *Actinobacteria* |
| BN-8 | *Streptomyces peucetius strain NBRC 100596* | AB249907 | 99% | *Actinobacteria* |
| BN-10 | *Streptomyces shaanxiensis strain CCNWHQ 0031* | FJ465151 | 99% | *Actinobacteria* |
| BN-11 | *Sinorhizobium meliloti 1021 strain 1021* | AL591688 | 99% | *α-Proteobacteria* |
| BN-12 | *Streptomyces peucetius strain NBRC 100596* | AB249907 | 99% | *Actinobacteria* |
| BH-1 | *Lysobacter niabensis strain GH34-4* | DQ462461 | 97% | *γ-Proteobacteria* |
| BH-2 | *Agromyces subbeticus strain Z33* | AY737778 | 99% | *Actinobacteria* |
| BH-4 | *Streptomyces peucetius strain NBRC 100596* | AB249907 | 99% | *Actinobacteria* |
| BH-5 | *Bacillus subtilis strain JCM 1465* | AB598736 | 99% | *Firmicutes* |
| BH-6 | *Arthrobacter pascens strain DSM 20545* | X80740 | 98% | *Actinobacteria* |
| BH-7 | *Bacillus halosaccharovorans strain E33* | HQ433447 | 99% | *Actinobacteria* |
| BH-9 | *Sinorhizobium meliloti 1021 strain 1021* | AL591688 | 99% | *α-Proteobacteria* |
| BH-10 | *Streptomyces diastato chromogenes strain ATCC 12309* | D63867 | 99% | *Actinobacteria* |
| CC-1 | *Microbacterium yannicii strain G72* | FN547412 | 98% | *Actinobacteria* |
| CC-2 | *Streptomyces prasinosporus strain NRRL B-12431* | DQ026655 | 99% | *Actinobacteria* |
| CC-3 | *Sinorhizobium meliloti 1021 strain 1021* | AL591688 | 99% | *α-Proteobacteria* |
| CC-4 | *Streptomyces peucetius strain NBRC 100596* | AB249907 | 99% | *Actinobacteria* |
| CC-5 | *FictiBacillus barbaricus strain V2-BIII-A2* | AJ422145 | 99% | *Firmicutes* |
| CC-6 | *Streptomyces coeruleofuscus strain NBRC 12757* | AB184840 | 99% | *Actinobacteria* |
| CC-7 | *Streptomyces tuirus strain NBRC 15617* | AB184690 | 99% | *Actinobacteria* |
| CC-8 | *Streptomyces coeruleofuscus strain ISP 5144* | AJ399473 | 99% | *Actinobacteria* |
| CC-9 | *Streptomyces cinereospinus strain NBRC 15397* | AB184648 | 99% | *Actinobacteria* |
| CC-10 | *PaeniBacillus xylanilyticus strain XIL14* | AY427832 | 98% | *Firmicutes* |
| CC-11 | *Bacillus litoralis strain SW-211* | AY608605 | 98% | *Firmicutes* |
| CN-1 | *Streptomyces variegatus strain NBRC 15462* | AB184688 | 99% | *Actinobacteria* |
| CN-2 | *Streptomyces avidinii strain NBRC 13429* | AB184395 | 99% | *Actinobacteria* |
| CN-3 | *Staphylococcus hominis subsp. novobiosepticus strain GTC 1228* | AB233326 | 99% | *Firmicutes* |
| CN-4 | *Streptomyces venezuelae strain NBRC 12595* | AB184836 | 99% | *Actinobacteria* |
| CN-5 | *Acinetobacter johnsonii strain ATCC 17909* | HE651920 | 99% | *γ-Proteobacteria* |
| CN-6 | *Streptomyces venezuelae strain NBRC 12595* | AB184836 | 99% | *Actinobacteria* |
| CN-7 | *PaeniBacillus xinjiangensis strain B538* | AY839868 | 99% | *Firmicutes* |
| CN-8 | *Pseudoxanthomonas wuyuanensis strain XC21-2* | JN247803 | 99% | *γ-Proteobacteria* |
| CN-10 | *Bacillus idriensis strain SMC 4352-2* | AY904033 | 99% | *Firmicutes* |
| CN-11 | *Sinorhizobium meliloti 1021 strain 1021* | AL591688 | 99% | *α-Proteobacteria* |
| CN-12 | *Streptomyces zaomyceticus strain NBRC 13348* | AB184346 | 99% | *Actinobacteria* |
| CN-13 | *Bacillus litoralis strain SW-211* | AY608605 | 99% | *Firmicutes* |
| CN-14 | *Sphingomonas mucosissima strain CP173-2* | AM229669 | 99% | *α-Proteobacteria* |
| CN-15 | *Serratia rubidaea strain JCM1240* | AB004751 | 99% | *γ-Proteobacteria* |
| CH-2 | *FictiBacillus phosphorivorans strain Ca7* | JX258924 | 99% | *Firmicutes* |
| CH-3 | *Streptomyces vastus strain NBRC 13094* | AB184307 | 97% | *Actinobacteria* |
| CH-4 | *Lechevalieria xinjiangensis strain R24* | DQ898283 | 99% | *Actinobacteria* |
| CH-5 | *Streptomyces rhizophilus strain JR-41* | HQ267989 | 98% | *Actinobacteria* |
| CH-6 | *Arthrobacter globiformis strain JCM 1332* | AB089841 | 99% | *Actinobacteria* |
| CH-7 | *Streptomyces peucetius strain NBRC 100596* | AB249907 | 99% | *Actinobacteria* |
| CH-8 | *Bacillus atrophaeus 1942 strain 1942* | CP002207 | 99% | *Firmicutes* |
